# Relevance of Wnt signaling for osteoanabolic therapy

**DOI:** 10.1186/2052-8426-2-22

**Published:** 2014-07-14

**Authors:** Timur A Yorgan, Thorsten Schinke

**Affiliations:** Department of Osteology and Biomechanics, University Medical Center Hamburg Eppendorf, Hamburg, 20246 Germany

**Keywords:** Bone remodeling, β-Catenin, Lrp5, Osteoblast, Sost, Wnt

## Abstract

The Wnt signaling pathway is long known to play fundamental roles in various aspects of embryonic development, but also in several homeostatic processes controlling tissue functions in adults. The complexity of this system is best underscored by the fact that the mammalian genome encodes for 19 different Wnt ligands, most but not all of them acting through an intracellular stabilization of β-catenin, representing the key molecule within the so-called canonical Wnt signaling pathway. Wnt ligands primarily bind to 10 different serpentine receptors of the Fzd family, and this binding can be positively or negatively regulated by additional molecules present at the surface of the respective target cells. One of these molecules is the transmembrane protein Lrp5, which has been shown to act as a Wnt co-receptor. In 2001, Lrp5, and thereby Wnt signaling, entered center stage in the research area of bone remodeling, a homeostatic process controlling bone mass, whose disturbance causes osteoporosis, one of the most prevalent disorders worldwide. More specifically, it was found that inactivating mutations of the human *LRP5* gene cause osteoporosis-pseudoglioma syndrome, a rare genetic disorder characterized by impaired bone formation and persistence of hyaloid vessels in the eyeballs. In addition, activating *LRP5* mutations were identified in individuals with osteosclerosis, a high bone mass condition characterized by excessive bone formation. Especially explained by the lack of cost-effective osteoanabolic treatment options, these findings had an immediate impact on the research regarding the bone-forming cell type, i.e. the osteoblast, whose differentiation and function is apparently controlled by Wnt signaling. This review summarizes the most important results obtained in a large number of studies, involving tissue culture experiments, mouse models and human patients. While there are still many open questions regarding the precise molecular interactions controlling Wnt signaling in osteoblasts, it is obvious that understanding this pathway is a key to optimize the therapeutic strategies for treating various skeletal disorders, including osteoporosis.

## Review

### Introduction

**Figure 1:**
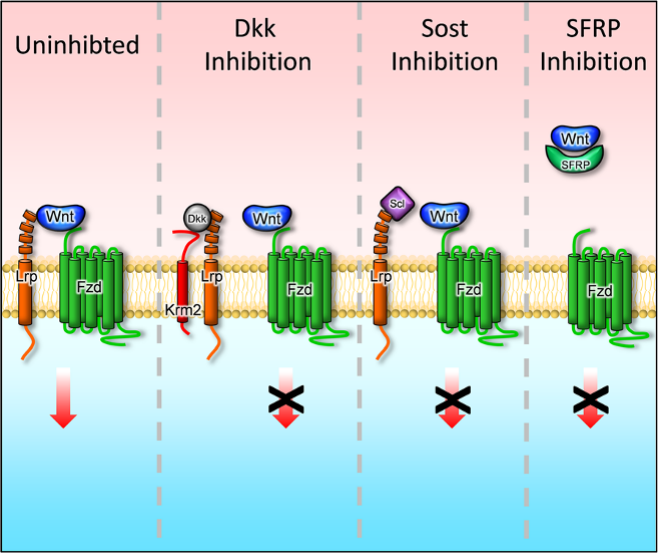
**Different mechanisms of Wnt singaling inhibition.** In an activated state (uninhibited) a Wnt molecule binds to a Fzd receptor and a co-receptor of the Lrp family. Dkk molecules interact with Krm receptors to form a ternary complex with Lrp co-receptors, thereby removing them from the activation complex [55]. Sclerostin (Scl) has been suggested to function in a similar way, yet its interaction with Lrp5 does not require Krm binding. In contrast, secreted Fzd-related proteins (Sfrps) sequester the activating Wnt ligand to antagonize Wnt signaling.

In 1982 the first Wnt gene was identified as a preferential integration site for MMTV (mouse mammary tumor virus) and originally termed *Int* [1]. This gene was found to represent the mouse homolog of the *Drosophila* gene *wingless*, and subsequently termed Wnt1 (**W**ingless and I**nt**-1) [2]. It is now known that the mammalian genome encodes for 19 different Wnt ligands, all of them characterized by a high number of conserved cysteine residues [3]. Although their precise molecular mode of action is variable, common properties, interaction partners and downstream signaling events have been identified, mostly triggered by *Drosophila* genetics, where many components of the canonical Wnt signaling pathway were originally identified. More specifically, although the Wnt ligands carry a classical N-terminal signal sequence, there is a specific endoplasmastic reticulum protein (Wntless) required to facilitate their secretion [4]. Another important step is a posttranslational cysteine palmitoylation, mediated by the enzyme Porcupine, which also causes poor solubility of the respective Wnt ligands, thus explaining their autocrine/paracrine mode of action [5, 6]. The primary Wnt receptors are Frizzled proteins, structurally belonging to the large family of serpentine receptors and encoded by 10 different *Fzd* genes in mice or humans [7, 8]. The Wnt-Fzd interaction is enhanced by single pass transmembrane co-receptors termed Arrow in *Drosophila* and Lrp5 (Low density lipoprotein receptor-related protein 5) or Lrp6 in the mammalian system [9–11]. The complexity of these ligand-receptor interactions is further enhanced by the existence of alternative Fzd/Lrp binding proteins, such as Norrin or R-Spondins [12, 13]. In addition, there are several extracellular molecules acting as Wnt signaling antagonists (Figure 1), such as soluble Fzd-related proteins (Sfrps) or members of the Dkk (Dickkopf) family, the latter ones binding to Lrp5/6 and inactivating their functions [14, 15]. One putative Wnt signaling antagonist, termed Sclerostin, was first identified by human genetics in individuals with increased bone formation, as discussed below [16, 17]. Given the fact that most of these mentioned protein families have several members, it is essentially impossible to establish a unifying concept for the mode of Wnt signaling activation in specific cellular settings.

### The Wnt signaling pathway

Based on this argument, it is not surprising that the intracellular signaling cascades triggered by Wnt binding to Fzd receptors are equally complex. In fact, various Wnt ligands have been shown to activate many different signaling pathways in a large number of distinct cell types [3, 7, 18]. Nevertheless, one particular pathway has emerged in *Drosophila* and mammalian cells as a major mediator of Wnt activation, and this pathway is known as canonical Wnt signaling [3, 18]. The key molecule within this process is β-catenin, a cytoplasmic protein that can enter the nucleus to regulate gene expression. In a non-activated state, β-catenin is mostly degraded by the proteasome, which requires the formation of a destruction complex containing the scaffold protein Axin2, the tumor suppressor APC and two serine/threonine kinases (CK1 and GSK3) that phosphorylate β-catenin to mark it for degradation [18]. Wnt binding to Fzd/Lrp receptors causes a rapid decomposition of the β-catenin destruction complex, mostly explained by Axin2 recruitment to the phosphorylated Wnt receptors. The stabilized non-phosphorylated β-catenin can enter the nucleus to interact with transcription factors of the Tcf/Lef family, thereby inducing transcription of specific target genes, one of them being *Axin2* [19, 20].

While canonical Wnt signaling is inducible by many different Wnt ligands, the efficacy of stimulation is variable. Moreover, some Wnt molecules have an entirely different mode of action and activate pathways summarized as non-canonical Wnt signaling [21, 22]. Although the precise mechanism of action remains to be clarified for most of the Wnt ligands, many researches have documented that for instance Wnt3a (a canonical Wnt ligand) and Wnt5a (a non-canonical Wnt ligand) have entirely different effects on cellular functions and gene expression. Whether these effects are generally true for various cell types in a physiologically relevant setting is one of the key questions for future research, especially since specific members of the Wnt pathway play fundamental roles for development and function of the organism. One of these molecules is Lrp5, whose mutation in mice and humans determines, how much bone matrix is built to form a stable skeleton.

### Osteoporosis, a major public health problem

**Figure 2:**
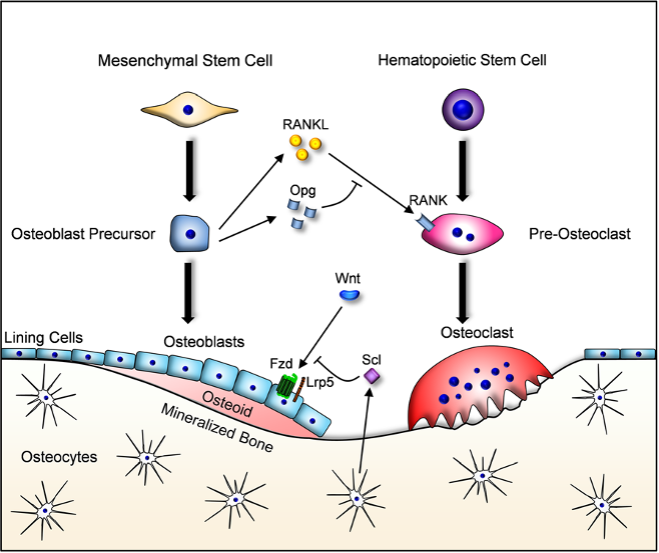
**Schematic presentation of the cell types involved in bone remodeling.** Bone-forming osteoblasts (left side) derive from mesenchymal progenitor cells and are arranged in large groups of cells simultaneously producing the bone matrix. This matrix is first non-mineralized (osteoid), before hydroxyapatite crystals get incorporated into the collagen fibrils to form mineralized bone. Some osteoblasts become embedded and differentiate into osteocytes, thereby forming a cellular network within the mineralized bone matrix. Bone-resorbing osteoclasts (right side) are derived from hematopoietic progenitors by cellular fusion. They are large multinucleated cells migrating along the bone surface to resorb it by two major mechanisms, i.e. extracellular acidification and secretion of matrix-degrading enzymes. The most important regulators of osteoclastogenesis (Rankl and Opg) and bone formation (Lrp5 and Sclerostin/Scl) are described in the text.

Osteoporosis is a systemic low bone mass disorder associated with an increased risk of skeletal fractures. It is considered as a major public health problem, not only because of its high prevalence (more than 200 million affected individuals worldwide), but also because skeletal fractures are associated with a high morbidity and mortality rate [23, 24]. The direct and indirect costs related to osteoporosis are currently estimated to be 38.7 billion € per year in the European Union, and since the yearly number of fractures is expected to double within the next 50 years, this socioeconomic problem will dramatically increase. This explains why there is an urgent need to define better options for prevention and/or treatment of osteoporosis, since the currently available strategies have several limitations. At the cellular level osteoporosis is explained by impaired bone remodeling, a physiologically relevant process mediated through the activities of two cell types, bone-resorbing osteoclasts and bone-forming osteoblasts. These two cell types are fundamentally different in terms of progenitor cells, mode of action and regulatory molecules controlling their differentiation and function (Figure 2). This explains why there are two distinct therapeutic options to treat osteoporosis, either osteoclast inhibition (anti-resorptive) or osteoblast activation (osteoanabolic).

More specifically, bone-forming osteoblasts derive from mesenchymal progenitor cells, which requires expression of specific transcription factors, such as Runx2 (Runt-related transcription factor 2) and Osx (Osterix) [25–27]. Once differentiated they act in large groups of cells that simultaneously produce an extracellular matrix primarily consisting of type I collagen, but also containing additional proteins, some of them bone-specific. This matrix is first non-mineralized and termed osteoid, but then gradually incorporates mineral in form of hydroxypapatite by mechanims, which are still not fully understood [28]. The same applies for the terminal step of osteoblast differentiation into a specialized cell type known as the osteocyte. These post-mitotic cells form a network within the mineralized bone matrix, and there is good evidence demonstrating their functions as orchestrators of bone remodeling, especially in response to mechanical stimuli [29]. In sharp contrast to osteoblasts, the bone-resorbing osteoclasts respresent a specialized hematopoietic cell type forming by fusion of monocyte/macrophage progenitors. This step is primarily regulated by the cytokine Rankl (Receptor activator of nuclear factor κB ligand), which is mostly produced by cells of the osteoblast lineage, thereby coupling bone resorption to bone formation. The pro-osteoclastogenic action of Rankl is physiologically inhibited by its decoy receptor Opg (Osteoprotegerin), a molecule expressed by osteoblasts in response to activated canonical Wnt signaling [30, 31]. Once differentiated the multinucleated osteoclasts resorb the mineralized bone matrix by two principial mechanisms, i.e. extracellular acidification and secretion of matrix-degrading enzymes.

For the treatment of osteoporosis, there are currently several types of anti-resorptive drugs available. These include the bisphophonates, a group of compounds non-specifically binding to mineralized bone, a monoclonal antibody neutralizing Rankl, SERMs (selective estrogen receptor modulators), or salmon calcitonin [32–34]. In contrast, there is only one type of osteoanabolic treatment available so far, daily injection of parathyroid hormone (PTH 1–84) or a PTH fragment (PTH 1–34) [35, 36]. Due to divergent cost-effectiveness of these therapeutic options, the vast majority of patients are currently treated with generically available bisphosphonates, i.e. alendronate or risedronate. Importantly however, it is conceivable to speculate that long-term blockade of bone resorption adversely affects skeletal integrity, as it interferes with the continuous renewal of the bone matrix. This is supported by an increasing number of case reports describing atypical long bone fractures in patients with bisphosphonate treatment for more than 5 years [37–41]. Moreover, since high bone mass due to impaired bone resorption (osteopetrosis) is associated with increased fracture risk, while the opposite is the case in states of high bone mass due to increased bone formation (osteosclerosis), one of the major goals of skeletal research is to identify novel target proteins for osteoanabolic medication [42]. This explains why it is of utmost clinical importance to identify molecules specifically regulating the activity of osteoblasts, and why the discovery of LRP5 as a gene affecting bone formation in humans was considered as a one of the biggest breakthroughs in the bone field ever.

### Lrp5, a Wnt co-receptor regulating bone formation

Given the above-described complexities of Wnt signaling and bone remodeling, it is quite useful that one particular statement is undoubted, namely that Lrp5 is a positive regulator of bone formation, not only in mice, but also in humans. The excitement about identifying the *LRP5* gene as a determinant of bone mass was initiated in the 1990s, where linkage analyses performed by several groups demonstrated the existence of a locus on human chromosome 11q13 segregating with two entirely different bone remodeling disorders [43, 44]. One disorder with autosomal recessive inheritance was osteoporosis pseudoglioma syndrome (OPPG), a condition characterized by low bone mass, skeletal fractures and persistence of embryonic eye vascularization causing blindness [45]. The other disorder, exhibiting autosomal dominant inheritance and usually termed high bone mass (HBM), was characterized by excessive bone formation, thickening of most bony structures and reduced fracture risk [44]. The fact that both conditions, at least with respect to bone formation, were essentially opposing each other raised the hypothesis that they were caused by mutations of the same gene, causing either loss or gain of function. A second hope was that the protein encoded by this gene could serve as a better target for osteoporosis treatment and prevention than the previously known regulators of bone formation.

Based on these arguments it was a big sensation that in 2001 inactivating mutations within the LRP5 gene were shown to cause OPPG, while gain-of-function mutations of LRP5 were shown to cause HBM [46–48]. Moreover, since the HBM mutations of LRP5 were found to be located within the extracellular region known to serve as a binding site for Wnt signaling antagonists of the Dkk family, there was an immediate and straightforward molecular explanation for both diseases. Most importantly however, since LRP5 was known to encode a transmembrane co-receptor for ligands of the Wnt family, the clinical and therapeutic relevance of these findings was tremendous. This explains why many researchers in the bone field became interested in studying the role of the Wnt signaling pathway for osteoblast differentiation and bone formation, and why many mouse models with impaired Wnt signaling have been analyzed for their skeletal phenotype (reviewed in [49]). Two of these models were the ones recapitulating OPPG and HBM. Here it was clearly shown that *Lrp5*-deficient mice displayed an osteoporotic phenotype solely explained by impaired bone formation, while bone resorption was unaffected [50]. Likewise, mice carrying an HBM mutation within the Lrp5 gene display osteosclerosis, i.e. high bone mass due to excessive osteoblast activity [51]. Moreover, since an *in vivo* anti-osteoanabolic function was also demonstrated for the Wnt signaling antagonist Dkk1, it was reasonable to speculate that the activity of Lrp5 is physiologically inhibited by binding of Dkk1 to the HBM region [52–55]. Although it is still possible that exactly this mechanism is the most relevant to explain the actions of Lrp5, this simplified concept has been challenged substantially, mostly explained by the complexities of the Wnt signaling pathway.

Since various findings obtained by analyzing genetically modified mouse models have been summarized in a recent review article [49], the present review will focus on two important issues regarding Lrp5-regulated signaling pathways and the relevant Lrp5 expression site. With respect to Wnt signaling, one obvious experiment was to inactivate β-catenin specifically in the two bone remodeling cell types. This was done by Cre-loxP-technology, where mice with a floxed β-catenin allele were crossed with different Cre-expressing mouse lines allowing cell-type specific inactivation. Here it was shown that deletion of β-catenin in cells of the osteoclast lineage results in increased osteoclastogenesis, while the deletion in mesenchymal osteoprogenitor cells causes an arrest of osteoblast differentiation and a shift towards chondrogenic differentiation [56–60]. Since Lrp5-deficiency however affects osteoblasts at a later stage of differentiation, the most important findings were obtained through the analysis of mice lacking β-catenin specifically in fully differentiated osteoblasts or osteocytes. Here it was found, in three different models, that β-catenin does not control osteoblast activity, as the bone formation rate was unaffected [30, 61, 62]. Instead, β-catenin inactivation in differentiated osteoblasts led to markedly increased osteoclastogenesis, molecularly explained by decreased production of Opg. These findings essentially rule out that Lrp5 controls bone formation by β-catenin-dependent signaling pathways, thus suggesting that Lrp5 either activates non-canonical Wnt signaling or functions in an entirely different manner.

A second highly relevant question is whether Lrp5 controls bone formation in a cell-autonomous manner, which is particularly important, since *Lrp5* is a ubiquitously expressed gene. Based on the finding that osteoblast proliferation is decreased in *Lrp5*-deficient mice, but not in *Lrp5*-deficient primary osteoblasts, one study attempted to identify the mode of *Lrp5* action in an unbiased approach, i.e. genome-wide expression analysis [63]. Here it was found that *Lrp5*-deficiency resulted in a dramatically increased expression of *Tph1* (Tryptophan hydroxylase 1), encoding the rate-limiting enzyme of peripheral serotonin biosynthesis. Although this differential expression was observable in osteoblasts, it was particularly pronounced in the duodenum, where the enterochromaffine cells are the major producers of peripheral serotonin. In a remarkable study the authors went on to demonstrate that Lrp5 controls bone formation in a serotonin-dependent manner, and most importantly they were able to show that both, inactivating or activating Lrp5 mutations only caused a bone phenotype, when present in the duodenum, while osteoblast-specific Lrp5 mutations did not affect skeletal remodeling [63]. Surprisingly however, another study using different targeting strategies, but aiming at the same question, came to an opposite conclusion. Here it was shown, also in a convincing manner, that Lrp5 activation or inactivation in osteocytes causes the expected bone formation phenotypes, whereas Lrp5 mutation in the duodenum had no effect on bone mass or circulating serotonin [64]. It is still remarkable that these two entirely different conclusions were made, and that the mode of Lrp5 action remains a matter of debate, even 13 years after the initial discovery as a major bone mass determinant in humans. What is clear however is that Lrp5 controls bone formation, while β-catenin in osteoblasts does not fulfil the same function.

### What is the molecular platform promoting bone formation together with Lrp5?

Although the relevant *Lrp5* expression site remains a matter of debate, there is one alternative explanation for the similar proliferation capacity of wildtype and *Lrp5*-deficient primary osteoblasts. In fact, assuming that Lrp5 acts as a Wnt co-receptor, a potential cell-autonomous defect of *Lrp5*-deficient osteoblasts may only be observed in the presence of a specific Wnt ligand. This is however not a trivial issue, since there is so far only limited knowledge about the nature and origin of the relevant Wnt ligand controlling bone formation. This implies for instance, that although Wnt3a administration to osteoblasts has been shown to regulate gene expression by inducing canonical Wnt signaling, it is purely speculative that such an effect is of any physiological relevance, especially in the context of Lrp5. Moreover, since osteogenesis and bone formation *in vivo* occurs in close proximity to various types of bone marrow cells, it is quite an important question, by which cell type a physiologically relevant Lrp5-interacting Wnt ligand is produced, and whether this particular cell type is present or absent in *ex vivo* cell culture systems. Another key question is related to the responsible Fzd receptor interacting with Lrp5. Although we have previously found that *Fzd9* is the only *Fzd* gene with differential expression during osteoblastogenesis *ex vivo*, and although *Fzd9*-deficient mice display reduced bone formation [65], it remains to be established, whether this particular receptor is a relevant interaction partner of Lrp5 and binds a specific Wnt ligand with osteoanabolic function. Again, this question is not easy to address, since the differences in primary structure between the 19 known Wnt molecules also translate into alternative modes of receptor interaction and downstream events.

In this regard it was again very helpful that inactivating mutations of specific Wnt molecules were found to be associated with low bone mass, either in mice or in humans [66–73]. Interestingly, the most evident osteoanabolic function of one particular Wnt ligand was only uncovered recently for the founding member of the family, i.e. Wnt1. More specifically, inactivating mutations of the human *WNT1* gene have been reported by different research groups in a large number of unrelated families with impaired bone formation [66, 72, 73]. The severity of the respective disorders ranged from fractures in early childhood, similar to osteogenesis imperfecta, to a moderate reduction of bone mineral density in adulthood, classified as early-onset osteoporosis. The large number of identified mutations segregating with the disease provides hallmark evidence for Wnt1 acting as a physiologically relevant osteoanabolic molecule. Surprisingly however, such a function has essentially been overlooked in mouse models with Wnt1 inactivation. Only recently, one group has carefully analyzed the phenotype of the *swaying* mice (*sw/sw*), carrying a spontaneous mutation of Wnt1 identified in 1991 [74]. These mice are primarily known for their neurologic deficits (which are not found in the patients), but their skeletal phenotype had not been studied until 2014. Here it was found that the *sw/sw* mice display a dramatically reduced bone formation rate causing severe osteoporosis with a fracture rate of 65% [75]. These remarkable findings raise the possibility that Wnt1 acts as ligand for Lrp5, and possibly Fzd9, which can now be addressed in appropriate mouse models and tissue culture experiments. From a therapeutic perspective it is extremely important to identify this molecular platform positively regulating bone formation, as potential drugs need to be developed against specific members of a given protein family.

### Sclerostin, a putative Lrp5-antagonist and an ideal drug target

In this regard it is consequential that the final paragraph of this review article will focus on a molecule that came out of nowhere in 2001 and that potentially acts as an Lrp5 antagonist. By definition, this molecule is highly relevant, as it was discovered by human genetics, again through analysis of families displaying osteosclerosis. The first report identified two inactivating mutations causing an autosomal recessive sclerosing bone dysplasia (sclerosteosis), and the thereby identified gene was termed *SOST* (Sclerostin) [16]. Immediately thereafter, another study identified a 52 kb deletion downstream of the *SOST* gene causing reduced transcription in individuals with van Buchem disease, a skeletal dysplasia with similarities to sclerosteosis [17]. At the time of discovery there was only little knowledge regarding the molecular action of Sclerostin (the protein encoded by the SOST gene), yet it was clear to be a secreted cysteine knot-containing protein with some homology to the DAN (differential screening selected gene abberative in neuroblastoma) family of Bmp (bone morphogenetic protein) antagonists. Although the mode of Sclerostin action is still not fully clarified more than 10 years thereafter, it is undoubted that this protein is primarily produced by osteocytes, and that it acts as an anti-osteoanabolic molecule. Not surprisingly, *Sost*-deficient mice display a remarkable high bone mass phenotype, whereas transgenic mice over-expressing *Sost* are osteoporotic [76, 77]. Since all of these phenotypes, in mice and humans, are caused by changes in bone formation, similar to what is known for Lrp5 mutations, it was immediately speculated that Sclerostin could act as an Lrp5 antagonist. This was first shown in 2005 and subsequently confirmed one year later, where the authors additionally found that the HBM mutations within the Lrp5 molecule interfere with Sclerostin binding [78–81]. Importantly, these data provided a unifying hypothesis for the function of the two different molecules, whose mutations cause osteosclerosis in humans. From then on Sclerostin was considered to represent a Wnt signaling antagonist binding to the HBM region of Lrp5 (Figure 1).

Although it is now speculated that this interaction is not solely responsible for the anti-osteoanabolic function of Sclerostin, as it also interacts with Lrp4, Lrp6 or BMPs [82–84], it is quite important to discuss the relevance of these findings in the present review article. In fact, if one only focuses on therapeutic relevance, it is not even necessary to fully understand the Sclerostin mechanism of action. What is mostly important, and this is undoubted, is that Sclerostin is a secreted protein that can be neutralized. This is why monoclonal antibodies against Sclerostin have been developed in order to test their application as an osteoanabolic drug. In 2014, i.e. 13 years after the identification of the first *SOST* mutations in individuals with sclerosteosis, the phase-II-clinical studies have been published [85]. Here it was found, that Sclerostin-specific antibodies, directly compared to two currently available treatment options (PTH and bisphosphonate), led to the strongest increase in bone mineral density after one year of administration by monthly injection. In addition, the first injections of this antibody led to a doubling of the serum concentrations of PINP (procollagen type I N-terminal propeptide), a biomarker of bone formation. Although this immediate osteoanabolic effect declined during the course of the one-year treatment, it is obvious that antagonizing Sclerostin holds great promise for the treatment and possibly prevention of osteoporosis. Whether the physiological action of Sclerostin is mediated by Lrp5 or Wnt signaling inhibition remains a question of basic research.

At that point it is also important to state that a similar approach is currently applied for Dkk1, whose inhibition might additionally be relevant to prevent bone destruction in a subset of cancer patients. More specifically, genome-wide expression analysis demonstrated elevated Dkk1 expression by myeloma tumor cells [86]. The potential therapeutic relevance of these findings was confirmed in animal experiments, where the administration of a Dkk1-neutralizing antibody attenuated the development of osteolytic lesions in immunodeficient mice engrafted with multiple myeloma cells [87–89]. Since Dkk1 inhibition has further been shown to attenuate erosive bone destruction in a mouse model of rheumatoid arthritis [90], it is highly relevant that Dkk-1 antibodies are under evaluation in clinical studies. Finally, since Sfrp1-deficiency has been shown to improve fracture healing in mice [91], it is reasonable to speculate that antagonizing this additional mechanism of Wnt signaling inhibition (Figure 1) is another therapeutic approach to improve skeletal integrity in patients.

Having such alternatives might in fact be extremely important, especially since the above-mentioned antibodies only cause a transient increase of bone formation biomarkers [85, 92]. Although one can only speculate about the underlying mechanisms so far (i.e. antibody development against the therapeutic antibodies, compensatory induction of other Wnt signaling components, or decreased expression of physiologically relevant osteoanabolic factors), this is surely a relevant problem to solve. In this context it is again important to come back to the overall complexities of bone remodeling regulation, especially regarding the bilateral crosstalk between osteoblasts and osteoclasts. Of note, it has been demonstrated that the osteoanabolic influence of PTH (1–84 and 1–34) is reduced by simultaneous treatment with the bisphosphonate alendronate [93, 94]. In contrast, a combination therapy with PTH (1–34 = Teriparatide) and a Rankl-specific antibody (Denosumab) was found to increase bone mineral density to a greater extent than the respective treatments alone [95]. With respect to Sclerostin inhibition it was found that pre-treatment or co-treatment with alendronate did not impair the effects of a Sclerostin antibody in ovariectomized rats [96]. Although this observation was principally confirmed in a recent clinical study comparing the effects of a Sclerostin antibody in naïve and bisphosphonate-treated individuals [92], it remains a matter of debate, whether it will be useful to combine Sost inhibition with specific anti-resorptives. While these questions have to be addressed in additional clinical studies, there is certainly a need to understand the cellular and molecular bases behind the present clinical observations and to follow alternative strategies to increase bone formation by activating Wnt singaling.

## Conclusions

Wnt signaling is an important pathway regulating many cell types, and after the discovery of *LRP5* mutations in individuals with altered bone formation Wnt signaling became highly relevant to understand bone remodeling and its disorders. Although Sclerostin antibodies are promising candidates for solving a huge clinical and socioeconomic problem, it is still useful to follow alternative approaches, especially since they are as promising. In this regard one key issue is surely to clearly define the interaction partners of Lrp5 that physiologically control bone formation. In particular, with respect to drug development, it is tremendously important to identify the specific members of the Wnt and Fzd protein families that are part of this molecular platform, together with Lrp5, possibly Sclerostin, and presumably some others. Given the speed of molecular genetic research in the last decades, this should however not be a major problem.

## References

[1] Nusse R, Varmus HE (1982). Many tumors induced by the mouse mammary tumor virus contain a provirus integrated in the same region of the host genome. Cell.

[2] Nusslein-Volhard C, Wieschaus E (1980). Mutations affecting segment number and polarity in Drosophila. Nature.

[3] Saito-Diaz K, Chen TW, Wang X, Thorne CA, Wallace HA, Page-McCaw A, Lee E (2013). The way Wnt works: components and mechanism. Growth Factors.

[4] Banziger C, Soldini D, Schutt C, Zipperlen P, Hausmann G, Basler K (2006). Wntless, a conserved membrane protein dedicated to the secretion of Wnt proteins from signaling cells. Cell.

[5] Zhai L, Chaturvedi D, Cumberledge S (2004). Drosophila wnt-1 undergoes a hydrophobic modification and is targeted to lipid rafts, a process that requires porcupine. J Biol Chem.

[6] Port F, Basler K (2010). Wnt trafficking: new insights into Wnt maturation, secretion and spreading. Traffic.

[7] Clevers H, Nusse R (2012). Wnt/beta-catenin signaling and disease. Cell.

[8] Wang HY, Liu T, Malbon CC (2006). Structure-function analysis of Frizzleds. Cell Signal.

[9] Tamai K, Semenov M, Kato Y, Spokony R, Liu C, Katsuyama Y, Hess F, Saint-Jeannet JP, He X (2000). LDL-receptor-related proteins in Wnt signal transduction. Nature.

[10] Pinson KI, Brennan J, Monkley S, Avery BJ, Skarnes WC (2000). An LDL-receptor-related protein mediates Wnt signalling in mice. Nature.

[11] Wehrli M, Dougan ST, Caldwell K, O'Keefe L, Schwartz S, Vaizel-Ohayon D, Schejter E, Tomlinson A, DiNardo S (2000). arrow encodes an LDL-receptor-related protein essential for Wingless signalling. Nature.

[12] Xu Q, Wang Y, Dabdoub A, Smallwood PM, Williams J, Woods C, Kelley MW, Jiang L, Tasman W, Zhang K, Nathans J (2004). Vascular development in the retina and inner ear: control by Norrin and Frizzled-4, a high-affinity ligand-receptor pair. Cell.

[13] Kazanskaya O, Glinka A, del Barco Barrantes I, Stannek P, Niehrs C, Wu W (2004). R-Spondin2 is a secreted activator of Wnt/beta-catenin signaling and is required for Xenopus myogenesis. Dev Cell.

[14] Bovolenta P, Esteve P, Ruiz JM, Cisneros E, Lopez-Rios J (2008). Beyond Wnt inhibition: new functions of secreted Frizzled-related proteins in development and disease. J Cell Sci.

[15] Semenov MV, Tamai K, Brott BK, Kuhl M, Sokol S, He X (2001). Head inducer Dickkopf-1 is a ligand for Wnt coreceptor LRP6. Curr Biol.

[16] Balemans W, Ebeling M, Patel N, Van Hul E, Olson P, Dioszegi M, Lacza C, Wuyts W, Van Den Ende J, Willems P, Paes-Alves AF, Hill S, Bueno M, Ramos FJ, Tacconi P, Dikkers FG, Stratakis C, Lindpainter K, Vickery B, Foernzler D, Van Hul W (2001). Increased bone density in sclerosteosis is due to the deficiency of a novel secreted protein (SOST). Hum Mol Genet.

[17] Balemans W, Patel N, Ebeling M, Van Hul E, Wuyts W, Lacza C, Dioszegi M, Dikkers FG, Hildering P, Willems PJ, Verheij JB, Lindpaintner K, Vickery B, Foernzler D, Van Hul W (2002). Identification of a 52 kb deletion downstream of the SOST gene in patients with van Buchem disease. J Med Genet.

[18] Clevers H (2006). Wnt/beta-catenin signaling in development and disease. Cell.

[19] Yan D, Wiesmann M, Rohan M, Chan V, Jefferson AB, Guo L, Sakamoto D, Caothien RH, Fuller JH, Reinhard C, Garcia PD, Randazzo FM, Escobedo J, Fantl WJ, Williams LT (2001). Elevated expression of axin2 and hnkd mRNA provides evidence that Wnt/beta -catenin signaling is activated in human colon tumors. Proc Natl Acad Sci U S A.

[20] Jho EH, Zhang T, Domon C, Joo CK, Freund JN, Costantini F (2002). Wnt/beta-catenin/Tcf signaling induces the transcription of Axin2, a negative regulator of the signaling pathway. Mol Cell Biol.

[21] Simons M, Mlodzik M (2008). Planar cell polarity signaling: from fly development to human disease. Annu Rev Genet.

[22] Kikuchi A, Yamamoto H, Sato A, Matsumoto S (2012). Wnt5a: its signalling, functions and implication in diseases. Acta Physiol (Oxf).

[23] Johnell O, Kanis JA (2006). An estimate of the worldwide prevalence and disability associated with osteoporotic fractures. Osteoporos Int.

[24] Kanis JA, Oden A, McCloskey EV, Johansson H, Wahl DA, Cooper C (2012). A systematic review of hip fracture incidence and probability of fracture worldwide. Osteoporos Int.

[25] Otto F, Thornell AP, Crompton T, Denzel A, Gilmour KC, Rosewell IR, Stamp GW, Beddington RS, Mundlos S, Olsen BR, Selby PB, Owen MJ (1997). Cbfa1, a candidate gene for cleidocranial dysplasia syndrome, is essential for osteoblast differentiation and bone development. Cell.

[26] Komori T, Yagi H, Nomura S, Yamaguchi A, Sasaki K, Deguchi K, Shimizu Y, Bronson RT, Gao YH, Inada M, Sato M, Okamoto R, Kitamura Y, Yoshiki S, Kishimoto T (1997). Targeted disruption of Cbfa1 results in a complete lack of bone formation owing to maturational arrest of osteoblasts. Cell.

[27] Nakashima K, Zhou X, Kunkel G, Zhang Z, Deng JM, Behringer RR, de Crombrugghe B (2002). The novel zinc finger-containing transcription factor osterix is required for osteoblast differentiation and bone formation. Cell.

[28] Rowe PS (2012). Regulation of bone-renal mineral and energy metabolism: the PHEX, FGF23, DMP1, MEPE ASARM pathway. Crit Rev Eukaryot Gene Expr.

[29] Bellido T (2014). Osteocyte-driven bone remodeling. Calcif Tissue Int.

[30] Glass DA, Bialek P, Ahn JD, Starbuck M, Patel MS, Clevers H, Taketo MM, Long F, McMahon AP, Lang RA, Karsenty G (2005). Canonical Wnt signaling in differentiated osteoblasts controls osteoclast differentiation. Dev Cell.

[31] Martin TJ (2013). Historically significant events in the discovery of RANK/RANKL/OPG. World J Orthop.

[32] Das S, Crockett JC (2013). Osteoporosis - a current view of pharmacological prevention and treatment. Drug Des Devel Ther.

[33] Smith MR, Egerdie B, Hernandez Toriz N, Feldman R, Tammela TL, Saad F, Heracek J, Szwedowski M, Ke C, Kupic A, Leder BZ, Goessl C (2009). Denosumab in men receiving androgen-deprivation therapy for prostate cancer. N Engl J Med.

[34] Cummings SR, San Martin J, McClung MR, Siris ES, Eastell R, Reid IR, Delmas P, Zoog HB, Austin M, Wang A, Kutilek S, Adami S, Zanchetta J, Libanati C, Siddhanti S, Christiansen C (2009). Denosumab for prevention of fractures in postmenopausal women with osteoporosis. N Engl J Med.

[35] Greenspan SL, Bone HG, Ettinger MP, Hanley DA, Lindsay R, Zanchetta JR, Blosch CM, Mathisen AL, Morris SA, Marriott TB (2007). Effect of recombinant human parathyroid hormone (1–84) on vertebral fracture and bone mineral density in postmenopausal women with osteoporosis: a randomized trial. Ann Intern Med.

[36] Neer RM, Arnaud CD, Zanchetta JR, Prince R, Gaich GA, Reginster JY, Hodsman AB, Eriksen EF, Ish-Shalom S, Genant HK, Wang O, Mitlak BH (2001). Effect of parathyroid hormone (1–34) on fractures and bone mineral density in postmenopausal women with osteoporosis. N Engl J Med.

[37] Shane E, Burr D, Ebeling PR, Abrahamsen B, Adler RA, Brown TD, Cheung AM, Cosman F, Curtis JR, Dell R, Dempster D, Einhorn TA, Genant HK, Geusens P, Klaushofer K, Koval K, Lane JM, McKiernan F, McKinney R, Ng A, Nieves J, O'Keefe R, Papapoulos S, Sen HT, van der Meulen MC, Weinstein RS, Whyte M (2010). Atypical subtrochanteric and diaphyseal femoral fractures: report of a task force of the American Society for Bone and Mineral Research. J Bone Miner Res.

[38] Odvina CV, Zerwekh JE, Rao DS, Maalouf N, Gottschalk FA, Pak CY (2005). Severely suppressed bone turnover: a potential complication of alendronate therapy. J Clin Endocrinol Metab.

[39] Visekruna M, Wilson D, McKiernan FE (2008). Severely suppressed bone turnover and atypical skeletal fragility. J Clin Endocrinol Metab.

[40] Bjorgul K, Reigstad A (2011). Atypical fracture of the ulna associated with alendronate use. Acta Orthop.

[41] Ang BF, Koh JS, Ng AC, Howe TS (2013). Bilateral ulna fractures associated with bisphosphonate therapy. Osteoporos Int.

[42] Rachner TD, Hadji P, Hofbauer LC (2012). Novel therapies in benign and malignant bone diseases. Pharmacol Ther.

[43] Gong Y, Vikkula M, Boon L, Liu J, Beighton P, Ramesar R, Peltonen L, Somer H, Hirose T, Dallapiccola B, De Paepe A, Swoboda W, Zabel B, Superti-Furga A, Steinmann B, Brunner HG, Jans A, Boles RG, Adkins W, van den Boogaard MJ, Olsen BR, Warman ML (1996). Osteoporosis-pseudoglioma syndrome, a disorder affecting skeletal strength and vision, is assigned to chromosome region 11q12-13. Am J Hum Genet.

[44] Johnson ML, Gong G, Kimberling W, Recker SM, Kimmel DB, Recker RB (1997). Linkage of a gene causing high bone mass to human chromosome 11 (11q12-13). Am J Hum Genet.

[45] Frontali M, Stomeo C, Dallapiccola B (1985). Osteoporosis-pseudoglioma syndrome: report of three affected sibs and an overview. Am J Med Genet.

[46] Boyden LM, Mao J, Belsky J, Mitzner L, Farhi A, Mitnick MA, Wu D, Insogna K, Lifton RP (2002). High bone density due to a mutation in LDL-receptor-related protein 5. N Engl J Med.

[47] Gong Y, Slee RB, Fukai N, Rawadi G, Roman-Roman S, Reginato AM, Wang H, Cundy T, Glorieux FH, Lev D, Zacharin M, Oexle K, Marcelino J, Suwairi W, Heeger S, Sabatakos G, Apte S, Adkins WN, Allgrove J, Arslan-Kirchner M, Batch JA, Beighton P, Black GC, Boles RG, Boon LM, Borrone C, Brunner HG, Carle GF, Dallapiccola B, De P (2001). LDL receptor-related protein 5 (LRP5) affects bone accrual and eye development. Cell.

[48] Little RD, Carulli JP, Del Mastro RG, Dupuis J, Osborne M, Folz C, Manning SP, Swain PM, Zhao SC, Eustace B, Lappe MM, Spitzer L, Zweier S, Braunschweiger K, Benchekroun Y, Hu X, Adair R, Chee L, FitzGerald MG, Tulig C, Caruso A, Tzellas N, Bawa A, Franklin B, McGuire S, Nogues X, Gong G, Allen KM, Anisowicz A, Morales AJ (2002). A mutation in the LDL receptor-related protein 5 gene results in the autosomal dominant high-bone-mass trait. Am J Hum Genet.

[49] Baron R, Kneissel M (2013). WNT signaling in bone homeostasis and disease: from human mutations to treatments. Nat Med.

[50] Kato M, Patel MS, Levasseur R, Lobov I, Chang BH, Glass DA, Hartmann C, Li L, Hwang TH, Brayton CF, Lang RA, Karsenty G, Chan L (2002). Cbfa1-independent decrease in osteoblast proliferation, osteopenia, and persistent embryonic eye vascularization in mice deficient in Lrp5, a Wnt coreceptor. J Cell Biol.

[51] Babij P, Zhao W, Small C, Kharode Y, Yaworsky PJ, Bouxsein ML, Reddy PS, Bodine PV, Robinson JA, Bhat B, Marzolf J, Moran RA, Bex F (2003). High bone mass in mice expressing a mutant LRP5 gene. J Bone Miner Res.

[52] Morvan F, Boulukos K, Clement-Lacroix P, Roman Roman S, Suc-Royer I, Vayssiere B, Ammann P, Martin P, Pinho S, Pognonec P, Mollat P, Niehrs C, Baron R, Rawadi G (2006). Deletion of a single allele of the Dkk1 gene leads to an increase in bone formation and bone mass. J Bone Miner Res.

[53] Ai M, Holmen SL, Van Hul W, Williams BO, Warman ML (2005). Reduced affinity to and inhibition by DKK1 form a common mechanism by which high bone mass-associated missense mutations in LRP5 affect canonical Wnt signaling. Mol Cell Biol.

[54] Li J, Sarosi I, Cattley RC, Pretorius J, Asuncion F, Grisanti M, Morony S, Adamu S, Geng Z, Qiu W, Kostenuik P, Lacey DL, Simonet WS, Bolon B, Qian X, Shalhoub V, Ominsky MS, Zhu Ke H, Li X, Richards WG (2006). Dkk1-mediated inhibition of Wnt signaling in bone results in osteopenia. Bone.

[55] Mao B, Wu W, Davidson G, Marhold J, Li M, Mechler BM, Delius H, Hoppe D, Stannek P, Walter C, Glinka A, Niehrs C (2002). Kremen proteins are Dickkopf receptors that regulate Wnt/beta-catenin signalling. Nature.

[56] Albers J, Keller J, Baranowsky A, Beil FT, Catala-Lehnen P, Schulze J, Amling M, Schinke T (2013). Canonical Wnt signaling inhibits osteoclastogenesis independent of osteoprotegerin. J Cell Biol.

[57] Wei W, Zeve D, Suh JM, Wang X, Du Y, Zerwekh JE, Dechow PC, Graff JM, Wan Y (2011). Biphasic and dosage-dependent regulation of osteoclastogenesis by beta-catenin. Mol Cell Biol.

[58] Otero K, Shinohara M, Zhao H, Cella M, Gilfillan S, Colucci A, Faccio R, Ross FP, Teitelbaum SL, Takayanagi H, Colonna M (2012). TREM2 and beta-catenin regulate bone homeostasis by controlling the rate of osteoclastogenesis. J Immunol.

[59] Hill TP, Spater D, Taketo MM, Birchmeier W, Hartmann C (2005). Canonical Wnt/beta-catenin signaling prevents osteoblasts from differentiating into chondrocytes. Dev Cell.

[60] Guo X, Day TF, Jiang X, Garrett-Beal L, Topol L, Yang Y (2004). Wnt/beta-catenin signaling is sufficient and necessary for synovial joint formation. Genes Dev.

[61] Holmen SL, Zylstra CR, Mukherjee A, Sigler RE, Faugere MC, Bouxsein ML, Deng L, Clemens TL, Williams BO (2005). Essential role of beta-catenin in postnatal bone acquisition. J Biol Chem.

[62] Kramer I, Halleux C, Keller H, Pegurri M, Gooi JH, Weber PB, Feng JQ, Bonewald LF, Kneissel M (2010). Osteocyte Wnt/beta-catenin signaling is required for normal bone homeostasis. Mol Cell Biol.

[63] Yadav VK, Ryu JH, Suda N, Tanaka KF, Gingrich JA, Schutz G, Glorieux FH, Chiang CY, Zajac JD, Insogna KL, Mann JJ, Hen R, Ducy P, Karsenty G (2008). Lrp5 controls bone formation by inhibiting serotonin synthesis in the duodenum. Cell.

[64] Cui Y, Niziolek PJ, MacDonald BT, Zylstra CR, Alenina N, Robinson DR, Zhong Z, Matthes S, Jacobsen CM, Conlon RA, Brommage R, Liu Q, Mseeh F, Powell DR, Yang QM, Zambrowicz B, Gerrits H, Gossen JA, He X, Bader M, Williams BO, Warman ML, Robling AG (2011). Lrp5 functions in bone to regulate bone mass. Nat Med.

[65] Albers J, Schulze J, Beil FT, Gebauer M, Baranowsky A, Keller J, Marshall RP, Wintges K, Friedrich FW, Priemel M, Schilling AF, Rueger JM, Cornils K, Fehse B, Streichert T, Sauter G, Jakob F, Insogna KL, Pober B, Knobeloch KP, Francke U, Amling M, Schinke T (2011). Control of bone formation by the serpentine receptor Frizzled-9. J Cell Biol.

[66] Keupp K, Beleggia F, Kayserili H, Barnes AM, Steiner M, Semler O, Fischer B, Yigit G, Janda CY, Becker J, Breer S, Altunoglu U, Grunhagen J, Krawitz P, Hecht J, Schinke T, Makareeva E, Lausch E, Cankaya T, Caparros-Martin JA, Lapunzina P, Temtamy S, Aglan M, Zabel B, Eysel P, Koerber F, Leikin S, Garcia KC, Netzer C, Schonau E (2013). Mutations in WNT1 cause different forms of bone fragility. Am J Hum Genet.

[67] Velazquez-Cruz R, Garcia-Ortiz H, Castillejos-Lopez M, Quiterio M, Valdes-Flores M, Orozco L, Villarreal-Molina T, Salmeron J (2014). WNT3A gene polymorphisms are associated with bone mineral density variation in postmenopausal mestizo women of an urban Mexican population: findings of a pathway-based high-density single nucleotide screening. Age (Dordr).

[68] Bennett CN, Longo KA, Wright WS, Suva LJ, Lane TF, Hankenson KD, MacDougald OA (2005). Regulation of osteoblastogenesis and bone mass by Wnt10b. Proc Natl Acad Sci U S A.

[69] Bennett CN, Ouyang H, Ma YL, Zeng Q, Gerin I, Sousa KM, Lane TF, Krishnan V, Hankenson KD, MacDougald OA (2007). Wnt10b increases postnatal bone formation by enhancing osteoblast differentiation. J Bone Miner Res.

[70] Stevens JR, Miranda-Carboni GA, Singer MA, Brugger SM, Lyons KM, Lane TF (2010). Wnt10b deficiency results in age-dependent loss of bone mass and progressive reduction of mesenchymal progenitor cells. J Bone Miner Res.

[71] Garcia-Ibarbia C, Perez-Nunez MI, Olmos JM, Valero C, Perez-Aguilar MD, Hernandez JL, Zarrabeitia MT, Gonzalez-Macias J, Riancho JA (2013). Missense polymorphisms of the WNT16 gene are associated with bone mass, hip geometry and fractures. Osteoporos Int.

[72] Laine CM, Joeng KS, Campeau PM, Kiviranta R, Tarkkonen K, Grover M, Lu JT, Pekkinen M, Wessman M, Heino TJ, Nieminen-Pihala V, Aronen M, Laine T, Kroger H, Cole WG, Lehesjoki AE, Nevarez L, Krakow D, Curry CJ, Cohn DH, Gibbs RA, Lee BH, Makitie O (2013). WNT1 mutations in early-onset osteoporosis and osteogenesis imperfecta. N Engl J Med.

[73] Pyott SM, Tran TT, Leistritz DF, Pepin MG, Mendelsohn NJ, Temme RT, Fernandez BA, Elsayed SM, Elsobky E, Verma I, Nair S, Turner EH, Smith JD, Jarvik GP, Byers PH (2013). WNT1 mutations in families affected by moderately severe and progressive recessive osteogenesis imperfecta. Am J Hum Genet.

[74] Thomas KR, Musci TS, Neumann PE, Capecchi MR (1991). Swaying is a mutant allele of the proto-oncogene Wnt-1. Cell.

[75] Joeng KS, Lee YC, Jiang MM, Bertin TK, Chen Y, Abraham AM, Ding H, Bi X, Ambrose CG, Lee BH (2014). The swaying mouse as a model of osteogenesis imperfecta caused by WNT1 mutations. Hum Mol Genet.

[76] Li X, Ominsky MS, Niu QT, Sun N, Daugherty B, D'Agostin D, Kurahara C, Gao Y, Cao J, Gong J, Asuncion F, Barrero M, Warmington K, Dwyer D, Stolina M, Morony S, Sarosi I, Kostenuik PJ, Lacey DL, Simonet WS, Ke HZ, Paszty C (2008). Targeted deletion of the sclerostin gene in mice results in increased bone formation and bone strength. J Bone Miner Res.

[77] Winkler DG, Sutherland MK, Geoghegan JC, Yu C, Hayes T, Skonier JE, Shpektor D, Jonas M, Kovacevich BR, Staehling-Hampton K, Appleby M, Brunkow ME, Latham JA (2003). Osteocyte control of bone formation via sclerostin, a novel BMP antagonist. EMBO J.

[78] Li X, Zhang Y, Kang H, Liu W, Liu P, Zhang J, Harris SE, Wu D (2005). Sclerostin binds to LRP5/6 and antagonizes canonical Wnt signaling. J Biol Chem.

[79] Semenov M, Tamai K, He X (2005). SOST is a ligand for LRP5/LRP6 and a Wnt signaling inhibitor. J Biol Chem.

[80] Ellies DL, Viviano B, McCarthy J, Rey JP, Itasaki N, Saunders S, Krumlauf R (2006). Bone density ligand, Sclerostin, directly interacts with LRP5 but not LRP5G171V to modulate Wnt activity. J Bone Miner Res.

[81] Semenov MV, He X (2006). LRP5 mutations linked to high bone mass diseases cause reduced LRP5 binding and inhibition by SOST. J Biol Chem.

[82] Kedlaya R, Veera S, Horan DJ, Moss RE, Ayturk UM, Jacobsen CM, Bowen ME, Paszty C, Warman ML, Robling AG (2013). Sclerostin inhibition reverses skeletal fragility in an Lrp5-deficient mouse model of OPPG syndrome. Sci Transl Med.

[83] Choi HY, Dieckmann M, Herz J, Niemeier A (2009). Lrp4, a novel receptor for Dickkopf 1 and sclerostin, is expressed by osteoblasts and regulates bone growth and turnover in vivo. PLoS One.

[84] Chang MK, Kramer I, Keller H, Gooi JH, Collett C, Jenkins D, Ettenberg SA, Cong F, Halleux C, Kneissel M (2014). Reversing LRP5-dependent osteoporosis and SOST deficiency-induced sclerosing bone disorders by altering WNT signaling activity. J Bone Miner Res.

[85] McClung MR, Grauer A, Boonen S, Bolognese MA, Brown JP, Diez-Perez A, Langdahl BL, Reginster JY, Zanchetta JR, Wasserman SM, Katz L, Maddox J, Yang YC, Libanati C, Bone HG (2014). Romosozumab in postmenopausal women with low bone mineral density. N Engl J Med.

[86] Tian E, Zhan F, Walker R, Rasmussen E, Ma Y, Barlogie B, Shaughnessy JD (2003). The role of the Wnt-signaling antagonist DKK1 in the development of osteolytic lesions in multiple myeloma. N Engl J Med.

[87] Yaccoby S, Ling W, Zhan F, Walker R, Barlogie B, Shaughnessy JD (2007). Antibody-based inhibition of DKK1 suppresses tumor-induced bone resorption and multiple myeloma growth in vivo. Blood.

[88] Fulciniti M, Tassone P, Hideshima T, Vallet S, Nanjappa P, Ettenberg SA, Shen Z, Patel N, Tai YT, Chauhan D, Mitsiades C, Prabhala R, Raje N, Anderson KC, Stover DR, Munshi NC (2009). Anti-DKK1 mAb (BHQ880) as a potential therapeutic agent for multiple myeloma. Blood.

[89] Heath DJ, Chantry AD, Buckle CH, Coulton L, Shaughnessy JD, Evans HR, Snowden JA, Stover DR, Vanderkerken K, Croucher PI (2009). Inhibiting Dickkopf-1 (Dkk1) removes suppression of bone formation and prevents the development of osteolytic bone disease in multiple myeloma. J Bone Miner Res.

[90] Diarra D, Stolina M, Polzer K, Zwerina J, Ominsky MS, Dwyer D, Korb A, Smolen J, Hoffmann M, Scheinecker C, van der Heide D, Landewe R, Lacey D, Richards WG, Schett G (2007). Dickkopf-1 is a master regulator of joint remodeling. Nat Med.

[91] Gaur T, Wixted JJ, Hussain S, O'Connell SL, Morgan EF, Ayers DC, Komm BS, Bodine PV, Stein GS, Lian JB (2009). Secreted frizzled related protein 1 is a target to improve fracture healing. J Cell Physiol.

[92] McColm J, Hu L, Womack T, Tang CC, Chiang AY (2014). Single- and multiple-dose randomized studies of blosozumab, a monoclonal antibody against sclerostin, in healthy postmenopausal women. J Bone Miner Res.

[93] Black DM, Greenspan SL, Ensrud KE, Palermo L, McGowan JA, Lang TF, Garnero P, Bouxsein ML, Bilezikian JP, Rosen CJ (2003). The effects of parathyroid hormone and alendronate alone or in combination in postmenopausal osteoporosis. N Engl J Med.

[94] Finkelstein JS, Hayes A, Hunzelman JL, Wyland JJ, Lee H, Neer RM (2003). The effects of parathyroid hormone, alendronate, or both in men with osteoporosis. N Engl J Med.

[95] Tsai JN, Uihlein AV, Lee H, Kumbhani R, Siwila-Sackman E, McKay EA, Burnett-Bowie SA, Neer RM, Leder BZ (2013). Teriparatide and denosumab, alone or combined, in women with postmenopausal osteoporosis: the DATA study randomised trial. Lancet.

[96] Li X, Ominsky MS, Warmington KS, Niu QT, Asuncion FJ, Barrero M, Dwyer D, Grisanti M, Stolina M, Kostenuik PJ, Simonet WS, Paszty C, Ke HZ (2011). Increased bone formation and bone mass induced by sclerostin antibody is not affected by pretreatment or cotreatment with alendronate in osteopenic, ovariectomized rats. Endocrinology.

